# Chemical composition and molecular structure of polysaccharide-protein biopolymer from *Durio zibethinus* seed: extraction and purification process

**DOI:** 10.1186/1752-153X-6-117

**Published:** 2012-10-14

**Authors:** Bahareh Tabatabaee Amid, Hamed Mirhosseini, Sanja Kostadinović

**Affiliations:** 1 Department of Food Technology, Faculty of Food Science and Technology, University Putra Malaysia, 43400 UPM Serdang, Selangor, Malaysia; 2 Faculty of Agriculture, University “Goce Delcev”, Krste Misirkov bb, 2000, Štip, Macedonia

**Keywords:** Biopolymer, *Durio zibethinus*, Molecular structure, Chemical structure, Carbohydrate, Essential amino acid, Fatty acid composition

## Abstract

**Background:**

The biological functions of natural biopolymers from plant sources depend on their chemical composition and molecular structure. In addition, the extraction and further processing conditions significantly influence the chemical and molecular structure of the plant biopolymer. The main objective of the present study was to characterize the chemical and molecular structure of a natural biopolymer from *Durio zibethinus* seed. A size-exclusion chromatography coupled to multi angle laser light-scattering (SEC-MALS) was applied to analyze the molecular weight (Mw), number average molecular weight (Mn), and polydispersity index (Mw/Mn).

**Results:**

The most abundant monosaccharide in the carbohydrate composition of durian seed gum were galactose (48.6-59.9%), glucose (37.1-45.1%), arabinose (0.58-3.41%), and xylose (0.3-3.21%). The predominant fatty acid of the lipid fraction from the durian seed gum were palmitic acid (C16:0), palmitoleic acid (C16:1), stearic acid (C18:0), oleic acid (C18:1), linoleic acid (C18:2), and linolenic acid (C18:2). The most abundant amino acids of durian seed gum were: leucine (30.9-37.3%), lysine (6.04-8.36%), aspartic acid (6.10-7.19%), glycine (6.07-7.42%), alanine (5.24-6.14%), glutamic acid (5.57-7.09%), valine (4.5-5.50%), proline (3.87-4.81%), serine (4.39-5.18%), threonine (3.44-6.50%), isoleucine (3.30-4.07%), and phenylalanine (3.11-9.04%).

**Conclusion:**

The presence of essential amino acids in the chemical structure of durian seed gum reinforces its nutritional value.

## Background

Durian (*Durio zibethinus*) is the most popular seasonal fruit in South East Asia countries, particularly Malaysia, Indonesia, Thailand, and Philippines [1,2]. The botanical taxonomy of durian brings to light many of the taxonomic problems. Initially, the genus *Durio* was created by Rumphius (1741) in his ‘Herbarium Amboinense’. Later, it was rendered into Linnaean by Adanson [3]. The type species *Durio zibethinus* is attributed to ‘Murr’. However, some researchers attributed the species to Linnaeus (L.) which appeared several times in early taxonomic literature. The earliest valid publication (Murray, 1774) also indicates that the botanical taxonomy of the species is referred to ‘*D. zibethinus* Linnaeus (L.). Willdenow (1800) also listed ‘*Durio zibethinus* as the species to Linnaeus (L.) [2,3].

There are hundreds of durian cultivars, but there are only 30 well recognized *Durio* species. At least nine species (i.e. D. *zibethinus,* D. *dulcis,* D. *grandiflorus,* D. *graveolens,* D. *kutejensis,* D. *lowianus,* D. *oxleyanus and* D. *testudinarum*) produce edible durian fruit [2]. Most cultivars have a common name including a code number such as Kop (D99), Chanee (D123), Tuan Mek Hijau (D145), Kan Yao (D158), D24 and D169. However, only *Durio zibethinus* is of economic importance and commercially grown cultivar. Only one-third of durian is edible; whereas the seeds (20-25%) are mostly thrown away after the consumption. Therefore, this crop waste can be a significant potential source of raw material useful for the development of value-added products (e.g., seed gum, flour and etc.).

The term “gum” is used to describe a group of naturally occurring polysaccharides and/or proteins originated from different sources (i.e. animal, plant and microbial). Natural plant gums are usually safe for oral consumption and are preferred over analogous synthetic gums due to their safety (non-toxic), low cost and availability [4]. Plant gums are usually heteropolysaccharide gums composed of simple hexoses and deoxy sugar units such as arabinose, galactose, glucose, mannose, xylose, uronic acids and etc. The chemical composition and molecular structure of polysaccharide plant gums play a significant role in their biological properties. In fact, the functional properties of polysaccharide plant gums are governed by the chemical composition, molecular weight, sequence of monosaccharide, configuration of glycoside linkages, and the position of glycoside linkages in the backbone and side chains [5].

The main goal of the current study was to investigate the effects of different extraction and purification methods on the chemical and molecular structure of durian seed gum. The chemical and molecular structure analysis were carried out by assessing the sugar composition, moisture, ash, lipid content, fatty acid composition, molecular weight (M_w_), number average molecular weight (Mn), and polydispersity index (Mw/Mn ratio). To the best of our knowledge, there is no similar study reporting the effect of different extraction and purification processes on the chemical and molecular structure of durian seed gum.

## Results and discussion

### Sugar composition of different crude durian seed gums

**Figure 1:**
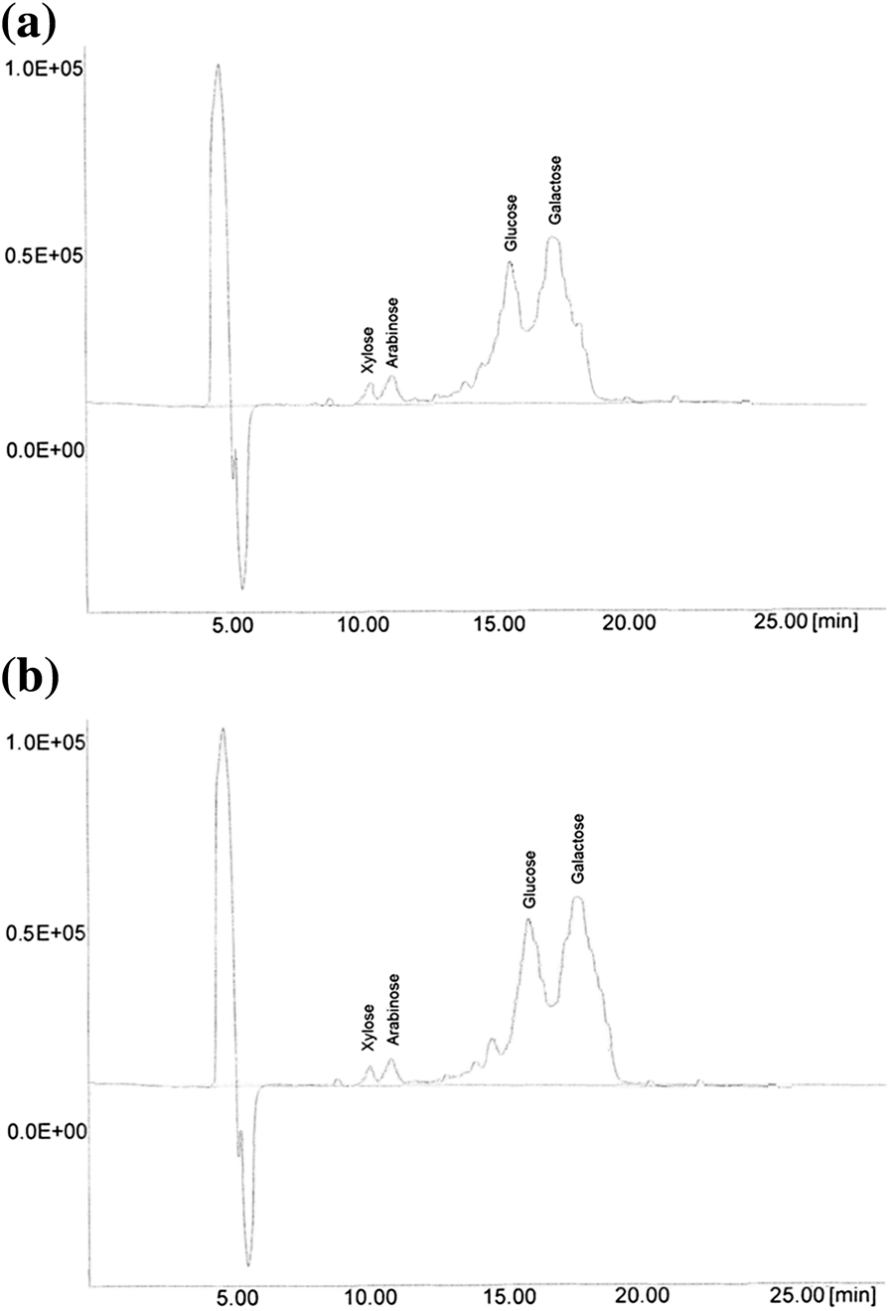
HPLC chromatograms showing the sugar profile of aqueous (a) and chemically (b)-extracted durian seed gums.

The sugar analysis revealed that D-galactose (54.4-58.2%) was the most abundant monosaccharide in the carbohydrate profile of aqueous- and chemically extracted gums from durian seed (Table 1, Figure 1). The results also demonstrated the presence of a high quantity of glucose (40.8-44.6%) in the carbohydrate profile of durian seed gum. This might be due to the accumulation of highly water soluble monosaccharide (i.e. glucose) during the extraction process. The presence of the high glucose content in the carbohydrate composition of durian seed gum might be also due to contamination from the seed coat. Amin and co-workers [6] also demonstrated that the glucose, galactose, and rhamnose were the main monosaccharide compositions in the molecular structure of crude durian seed gum. However, the current study revealed the presence of a low percentage of rhamnose in the chemical structure of durian seed gum. This difference might be due to different extraction methods, different experimental conditions of the carbohydrate analysis. Dawkins and Nnanna [7] also reported a high percentage of glucose (98.4%) in the gum from oat seed. As reported by Palanuvej et al. [8], galactose and glucose were the main monosaccharide in the chemical composition of glucomannan from *Litsea glutinosa* leaves, *Hibiscus esculentus* and *Scaphium scaphigerum* fruits, *Ocimum canum*, *Plantago ovata* and *Trigonella foenum-graecum* seeds. As shown in Table 1, the percentage of glucose and galactose of durian seed gum was higher than that of Yanang gum [9], locust bean gum [10], malva nut gum [11], and Prosopis seed gum [12]. Conversely, it showed a lower percentage of arabinose and xylose than Yanang gum, locust bean gum, and Malva nut gum (Table 1).

**Table 1:** Comparison between sugar composition of durian seed gum and other plant based gums

**Plant gum**	**Monosaccharide composition**					
	**Rham**	**Xyl**	**Arab**	**Glu**	**Gal**	**Man**
Durian seed gum ^a^	trace	0.4 ± 0.1	0.6 ± 0.0	40.8 ± 3.9	58.2 ± 3.2	-
Durian seed gum ^b^	trace	0.3 ± 0.0	0.8 ± 0.1	44.6 ± 2.8	54.4 ± 3.5	-
Durian seed gum ^c^	21	-	-	70	8	-
Yanang gum ^d^	0.5	72.9	7.7	11.0	8.4	-
Locust bean gum ^e^	0.2 ± 0.1	0.6 ± 0.1	1.9 ± 0.1	4.1 ± 0.1	14.6 ± 0.2	51.9 ± 0.5
Malva nut gum ^f^	29.4 ± 0.1	2.1 ± 0.1	31.9 ± 0.2	2.7 ± 0.2	29.2 ± 0.2	4.8 ± 0.3
Prosopis seed gum ^g^	-	-	2.5 ± 0.2	13.9 ± 1.2	27.3 ± 0.8	56.3 ± 0.8

Data presented are the mean value ± standard deviation, *Rham* Rhamnose, *Xyl* Xylose, *Arab* Arabinose, *Glu* Glucose, *Gal* Galactose, *Man* Mannose, ^a^ Crude aqueous durian seed gum (current study), ^b^ Crude chemically-extracted durian seed gum (current study), ^c^ Amin et al. [6], ^d^ Singthong et al. [9], ^e^ Dakia et al. [10], ^f^ Somboonpanyakul et al. [11], ^g^ Ibaňez and Ferrero [12].

The results also showed the presence of the low amount of xylose and arabinose and trace amount of rhamnose in the chemical structure of aqueous- and chemically extracted gums from durian seed (Table 1, Figure 1). Palanuvej et al. [8] also reported the presence of minor content of xylose and arabinose in the chemical composition of glucomannan from *Litsea glutinosa* leaves, *Hibiscus esculentus* and *Scaphium scaphigerum* fruits. As stated by previous researchers [13], arabinose is a pentose monosaccharide which contributes to the molecular structure of various plant gums. Low level of arabinose is normally occurring as a free sugar in the side chain of polysaccharide gums; where xylose are mostly present in the backbone of polysaccharide gum (arabinoxylan) [13]. According to Da Silva and Gonçalves [14], the presence of a minor content of arabinose, xylose and glucose could be attributed to a more complex polysaccharide composition. They also demonstrated that this may be due to contaminants proceeding from the seed coat. As stated by León De Pinto and co-researchers [15], arabinose and xylose are positioned as terminal and terminal residues in the chemical structure of *Acacia tortuosa*, respectively. In general, the chemically-extracted durian seed gum had a higher percentage of arabinose and glucose as well as a lower percentage of xylose and galactose than the aqueous-extracted durian seed gum (Table 1). Both durian seed gums had a lower percentage of xylose than flaxseed gum reported by previous researchers [16]. Although, the carbohydrate analysis of durian seed gum confirmed the presence of high galactose content, but it did not reveal the presence of mannose in the carbohydrate composition of durian seed gum (Table 1). In fact, durian seed gum does not seem to have mannose; therefore, it is classified as a glucogalactan, not galactomannan or arabinogalactan.

### Sugar composition of different purified durian seed gums

**Figure 2:**
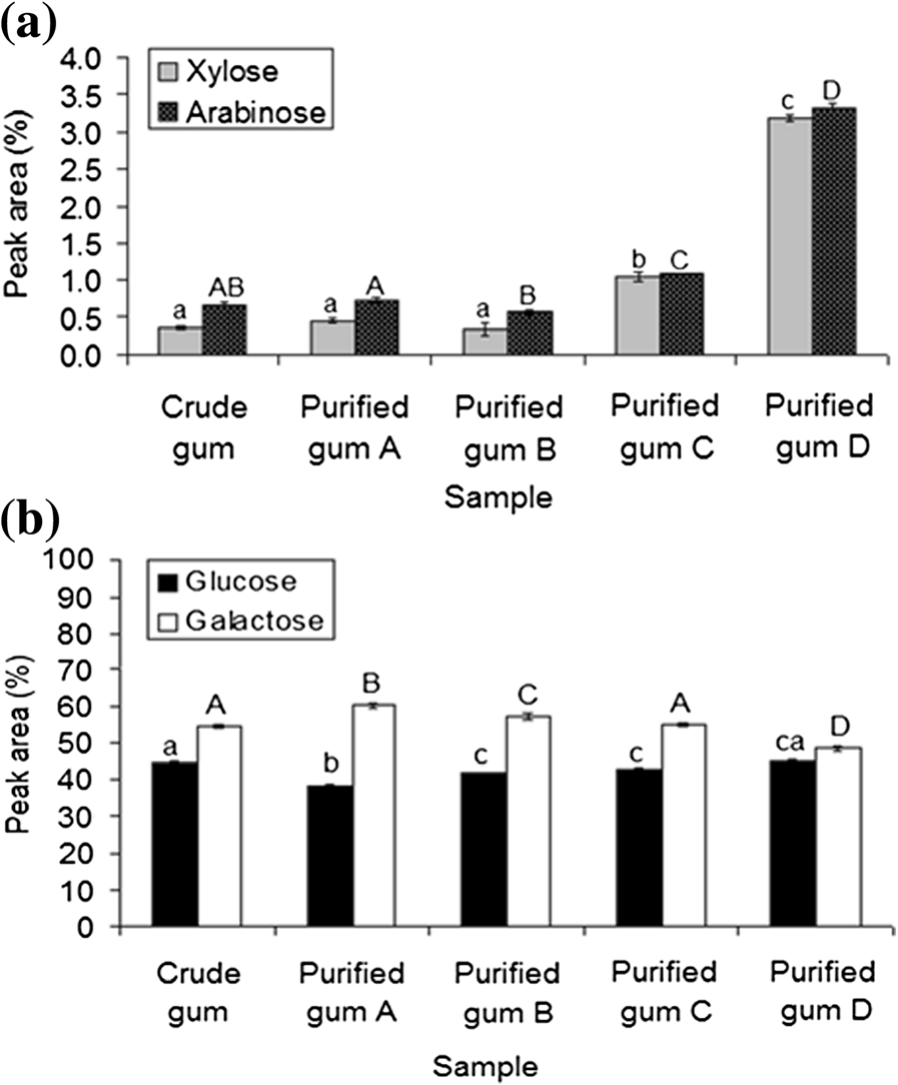
The percentage of xylose and arabinose (a) as well as glucose and galactose (b) in crude and different purified durian seed gums (A: isopropanol and ethanol; B: isopropanol and acetone; C: saturated barium hydroxide; D: Fehling solution); Mean ± standard deviation; a-c: Significant (p < 0.05) difference among samples in terms of xylose and glucose; A-D: Significant (p < 0.05) difference among samples in terms of arabinose and galactose.

The results showed that the purification process significantly (p < 0.05) affected the carbohydrate composition of crude durian seed gum (Figure 2). However, the significant effect of the purification process on the carbohydrate profile seems to be dependent upon the process condition. The precipitation using Fehling solution (Method D) caused the most significant effect of on the percentage of arabinose; while both purification method A and B had the least effect on the arabinose content. The results showed that the purified gum D and B had the highest and lowest percentage of arabinose among all samples. The purification using saturated barium hydroxide (Method C) also induced the significant (p < 0.05) effect on the percentage of arabinose (Figure 2a).

The current study revealed that the purification using Fehling solution resulted in the highest significant (p < 0.05) changes in the monosaccharide composition (i.e. xylose and arabinose) of durian seed gum (Figure 2). As stated by previous researchers [17], Fehling solution containing 3M NaOH can promote the chain degradation of polysaccharide, thereby affecting the monosaccharide composition of the gum. On the other hand, the chemical purification using isopropanol and ethanol (Method A) and isopropanol and acetone (Method B) did not significantly change the percentage of xylose present in the carbohydrate composition of durian seed gum (Figure 2a). However, both purified gums A and B had the lower xylose content than the crude gum (Figure 2a). Da Silva and Gonçalves [14] also reported that the purified locust bean gum showed a lower content of xylose than its crude gum. The significant changes in xylose content may significantly influence the rheological properties of the gum. As stated by previous researches [18], xylose content reflects the relative amount of neutral polysaccharides, which enhances rheological properties of the gum by increasing the shear thinning behavior and weak-gel properties.

As shown in Figure 2b, the purified gum D and A showed the highest and lowest percentage of glucose among all purified gums. In general, all purification processes (except for Method D) significantly (p < 0.05) reduced the percentage of glucose in durian seed gum. The level of glucose reduction was dependent upon the purification technique as follows: purified gum A > purified gum B > purified gum C > crude gum > purified gum D (Figure 2b). Previous researchers [14,19,20] also reported the glucose reduction after purifying the crude locust bean gum, fenugreek gum and guar gum, respectively.

As shown in Figure 2b, the purified gum A (isopropanol and ethanol) and B (isopropanol and acetone) showed a higher percentage of galactose than the crude gum. Previous researchers [19] also reported the similar observation for the crude and purified fenugreek gum. They found that the purified fenugreek gum had a higher percentage of galactose than the crude fenugreek gum. The results indicated that the purified gum A and D showed the highest and lowest percentage of galactose among all samples (Figure 2b). Our previous study [21] reported the significant (p < 0.05) different rheological properties and viscoelastic behavior of the crude and purified gums. This might be explained by the significant (p < 0.05) effect of the purification process on the carbohydrate composition of durian seed gum.

### Moisture and ash content

The chemical extraction method resulted in lower moisture content than the aqueous extraction method. On the other hand, there was a significant (p < 0.05) different between the moisture content of the crude and purified durian seed gums. In general, all purification methods led to reduce the moisture content as compared to both crude gums (Table 2). The purified gum A and C had the highest and lowest significant moisture content among all purified gum. However, the purified gum A had higher moisture content than the crude gum (Table 2). Durian seed gum had a relatively high content of moisture (20.5-26.8%) in both crude and purified forms (Table 2). This value was greater than the moisture content reported for corn fiber gum (4.0-5.9%) [22], guar gum (7.36%), locust bean gum (7.92%), and gum karaya (9.43%) [23], acacia glomerosa gum (11.29) [24], grewia gum (10.6- 18.8) [25], and Baobab leaves gum (7.8-8.0) [26] (Table 3).

**Table 2:** Moisture, ash and lipid content of durian seed gum

**Test**	**Crude gum (Aqueous)**	**Crude gum (Chemical)**	**Purified gum A**	**Purified gum B**	**Purified gum C**	**Purified gum D**
Moisture %	26.8 ± 1.09^a^	24.6 ±1.22^ab^	23.2 ± 1.03^b^	22.8 ± 0.79^bc^	20.5 ± 0.35^c^	21.7 ± 1.13^bc^
Total ash %	32.8 ± 1.57^a^	34.3 ± 1.78^a^	20.6 ± 0.89^b^	23.4 ± 1.34^b^	15.8 ± 1.13^c^	12.1 ± 0.94^d^
Soluble ash %	1.7 ± 0.03^a^	1.5 ± 0.01^a^	0.9 ± 0.00^b^	1.0 ± 0.14^b^	0.7 ± 0.03^c^	0.5 ± 0.06^d^
Lipid %	1.92 ± 0.07^a^	0.78 ± 0.11^b^	0.14 ± 0.04^c^	0.16 ± 0.02^c^	0.21 ± 0.06^d^	0.19 ± 0.03^d^

Data presented are the mean value ± standard deviation, *A *isopropanol and ethanol, *B *isopropanol and acetone, *C *saturated barium hydroxide, *D *Fehling solution, ^a-d ^significant differences at 95% confident level.

**Table 3:** Moisture, ash, and molecular weight (Mw) of various plant gums

**Sample**	**Moisture %**	**Ash %**	**Soluble ash%**	**Mw**
Acacia glomerosa gum ^1^	11.29	7.98	-	0.9 × 10^5^
Corn fiber gum ^2^	4.0-6.6	4.0-5.9	-	-
Grewia gum ^3^	10.6- 18.8	6.1 -6.3	3.4 -3.8	-
Okra fruit gum ^4^	9.35-9.37	4.81-5.95	-	-
Baobab leaves gum ^4^	7.8-8.0	8.8-9.88	-	> 1.0 ×10^5^

^1^ Gundidza et al. [24]; ^2^ Yadav et al. [22]; ^3^ Nep and Conway [25]; ^4^ Woolfe et al. [26].

The total ash is a useful figure for determining the characterization and purity of the gum [27]. This parameter gives an indication of the degree of mineral interaction in the structure which contributes to the functional properties of the polysaccharide gum. The lower ash content is associated with higher purity degree [27]. The results indicated that the purification process significantly (p < 0.05) decreased the ash content of durian seed gum. On the other hand, the purification significantly (p < 0.05) affected the soluble ash content as compared to the crude gum (Table 2). The degree of changes depended on the purification condition (Table 2). The current study revealed that durian seed gum had a relatively high content of ash (12.1 ± 34.3%). This value was higher than the ash content reported for Africana seed gum (3.63-3.65%) [24], corn fiber gum (4.0-5.9) [28], gum Arabic (1.2%), guar (11.9%) [16], grewia gum (6.1 -6.3%) [25], okra fruit gum (4.81-5.95%), and baobab leaves gum (8.8-9.88%) [26] (Table 3). Amin and co-researchers [6] also reported a relatively high total ash (29.8%) in durian seed gum. The results also indicated that both crude and purified durian seed gums had relatively low content of soluble ash content, ranging from 0.5 to 1.7% (Table 2). The purified gum D followed by purified gum C had the lowest content of soluble ash; while the aqueous and chemically extracted crude gums provided the highest soluble ash among all samples (Table 2). This might indicate the efficiency of all purification processes to reduce the gum impurities.

### Lipid content and fatty acid composition

The results indicated that different crude and purified durian seed gums had significant (p < 0.05) different lipid content, ranging from 0.19 to 1.92% (Table 2). A comparative lipid analysis of different crude and purified durian seed gums showed that the purification process significantly (p < 0.05) reduced the lipid content, thus enhancing the gum purity (Table 2). However, none of purification techniques enabled to completely remove the lipid fraction present in the chemical structure of the crude durian seed gum. This could be explained by the fact that the lipid fraction might be a part of chemical structure of durian seed gum. Yadav and co-researchers [22] reported the presence of the lipid fraction as a part of the molecular structure of gum Arabic, inducing the emulsifying activity. In addition, the presence of the lipid fraction in the chemical structure of many polysaccharide gums have been reported by previous researchers [22,28,29].

As mentioned earlier, both aqueous and chemically extracted durian seed gum contained a relatively higher content of lipid fraction than the purified gums (A-D). Our previous study [30] revealed that the crude gum from durian seed showed the interfacial activity in oil/water (o/w) emulsion system. This might be due to the presence of trace amount of the hydrophobic lipid fraction along with the hydrophilic polysaccharide fraction present in the chemical structure of durian seed gum. However, the emulsifying activity of durian seed gum might be also due to the presence of the protein fraction present in the chemical structure of durian seed gum may also contribute to its emulsifying activity [30]. The chemically-extracted crude gum showed a significant (p < 0.05) lower fat content (0.78 ± 0.11) than the aqueous crude gum (1.92 ± 0.07). This might be due to the defatting process occurred during the chemical extraction process. In fact, durian seed was defatted and discolored by using organic solvent during the chemical gum extraction, thus more efficiently reducing the lipid fraction impurity than the aqueous extraction process. This indicates the less efficiency of aqueous extraction technique than the chemical extraction method to reduce the hydrophobic impurities (i.e. lipid fraction).

On the other hand, the significant different between the lipid content of aqueous- and chemically-extracted durian seed gums might be responsible for their significant different levels of solubility. The results indicated that the precipitation using isopropanol and ethanol (Method A) looks to be the most efficient purification technique for the removal of the hydrophobic lipid fraction from the crude durian seed gum (Table 2). Conversely, the precipitation using saturated barium hydroxide seems (Method C) seems to be the least efficient purification technique for reducing the hydrophobic impurity (i.e. lipid fraction) of the crude durian seed gum (Table 2). As also shown in Table 2, the purified gum A and C had the lowest and highest content of the lipid fraction among all purified gums.

**Figure 3:**
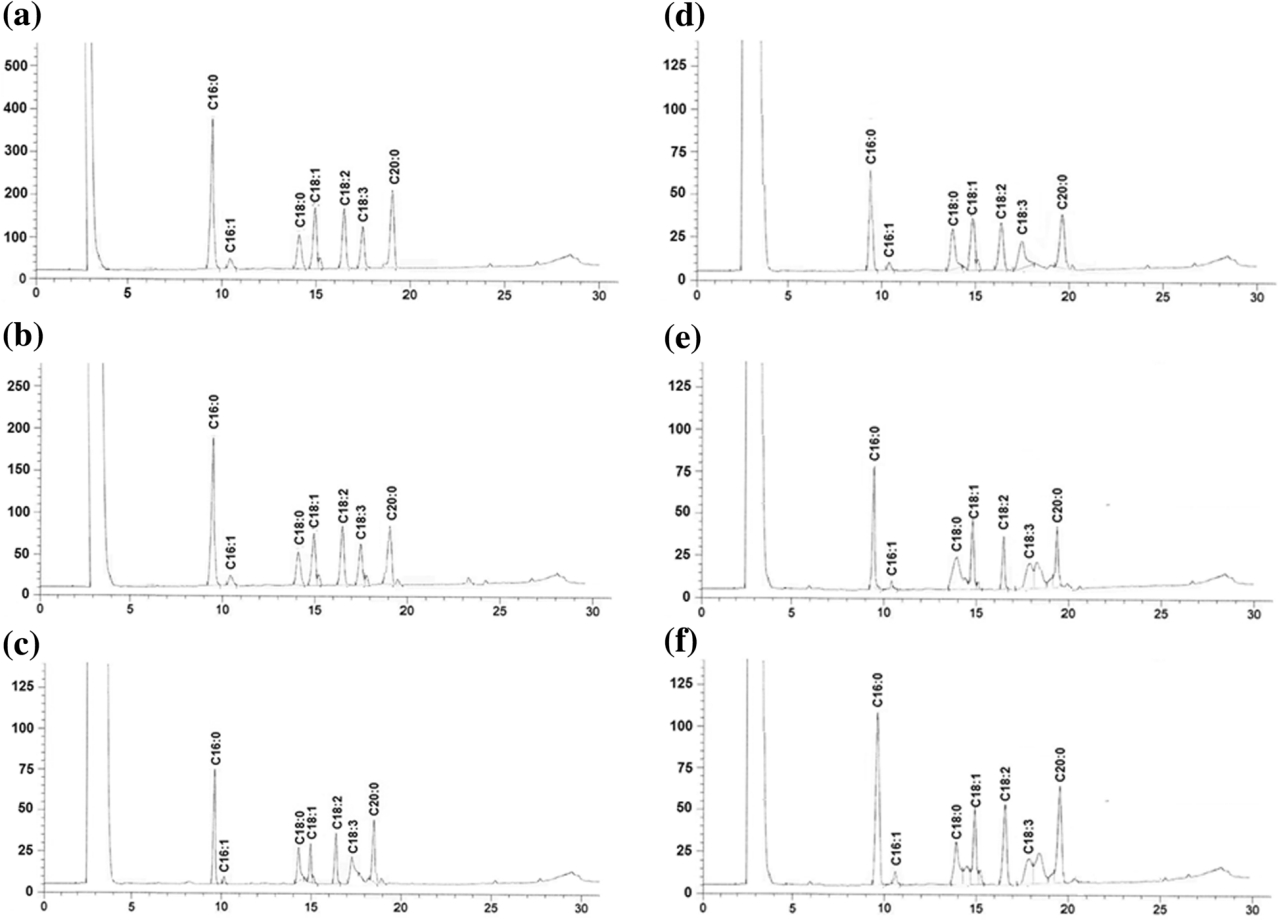
Fatty acid profile of the lipid fraction from (a) aqueous crude gum, (b) chemical crude gum, and (c-f) purified seed gums (a-d); C16:0 (palmitic acid), C16:1 (palmitoleic acid), C18:0 (stearic acid), C18:1 (oleic acid), C18:2 (linoleic acid), C18:2 (linolenic acid), and C20:0 (arachidic acid).

Figure 3 shows the fatty acid composition of the lipid fraction from (a) aqueous extracted crude gum, (b) chemically-extracted crude gum, (c) purified seed gum A, (d) purified seed gum B, (e) purified seed gum C, and (f) purified seed gum D. The predominant fatty acid of the lipid fraction obtained from the crude and durian seed gums were palmitic acid (C16:0), palmitoleic acid (C16:1), stearic acid (C18:0), oleic acid (C18:1), linoleic acid (C18:2), linolenic acid (C18:2), and arachidic acid (C20:0) (Table 4). Previous researchers [28] also reported the presence of palmitic acid (C16:0) and oleic acid (C18:1) in the lipid fraction extracted from corn fibre gum. The current study revealed that the lipid fraction from durian seed had a higher amount of saturated fatty acid (SFA) (C18:0, C18:0 and C20:0) rather than unsaturated fatty acid (C16:1, C18:1, C18:2 and C18:3).

**Table 4:** Fatty acid composition present in the chemical structure of durian seed gum

**Gum**	**Fatty acid**^**a**^						
	**C16:0**	**C16:1**	**C18:0**	**C18:1**	**C18:2**	**C18:3**	**C20:0**
Crude gum ^1^	642 ± 57^a^	52 ± 9^a^	287 ± 17^a^	374 ± 28^a^	346 ± 48^a^	75 ± 11^a^	423 ± 39^a^
Crude gum ^2^	337 ± 42^b^	33 ± 2^b^	138 ± 12^b^	169 ± 23^b^	158 ± 26^b^	36 ± 4^b^	151 ± 18^b^
Purified gum A	141 ± 36^cd^	15 ± 3^c^	76 ± 9^c^	63 ± 11^c^	76 ± 8^c^	14 ± 3^c^	62 ± 5^c^
Purified gum B	116 ± 19^c^	21 ± 5^c^	93 ± 13^cd^	107 ± 10^d^	103 ± 16^bc^	17 ± 3^c^	76 ± 12^c^
Purified gum C	158 ± 31^cd^	11 ± 3^c^	108 ± 16^d^	99 ± 14^cd^	95 ± 13^c^	25 ± 6^bc^	69 ± 7^c^
Purified gum D	203 ± 46^d^	24 ± 4^c^	101 ± 15^cd^	141 ± 17^bd^	146 ± 21^b^	31 ± 5^b^	137 ± 23^b^

^a ^In μg/g of the dry weight of samples; Data presented are the mean value ± standard deviation; ^1^: Aqueous crude gum; ^2^: Chemically extracted crude gum; C16:0, palmitic acid; C18:0, stearic acid; C18:1, oleic acid; C18:2, linoleic acid; C18:3, linolenic acid; A: isopropanol and ethanol; B: isopropanol and acetone; C: saturated barium hydroxide; D: Fehling solution.

As shown in Table 4, the aqueous-extracted gum seems to have the highest content of saturated fatty acid (SFA) (C18:0, C18:0 and C20:0), monounsaturated fatty acid (MUFA) (C16:1 and C18:1), and polyunsaturated fatty acid (PUFA) (C18:2 and C18:3) among all crude and purified gums (A-D). In most cases, the purified gum D had the highest percentage of SFA, MUFA and PUFA among all purified sample; while the purified gum A showed the lowest significant (p < 0.05) content of C18:0, C18:1, C18:2, C18:3 and C20:0 among all samples (Table 4). The purification using isopropanol and ethanol (Method A) and isopropanol and acetone (Method B) seems to have more efficiency than the precipitation using saturated barium hydroxide (Method C) and Fehling solution (Method D) for reducing SFA, MUFA and PUFA present in the chemical structure of durian seed gum.

Vinod and co-researchers [31] reported the presence SFA such as capric acid (C10:0), lauric acid (C12:0), myristic acid (C14:0), palmitic acid (C16:0), stearic acid (C18:0), arachidic acid (C20:0), behenic acid (C22:0), and lignoceric acid (C24:0) in the fatty acids composition of gum kondagogu. They found that stearic acid (25.4 ± 1.54 lg/g) was the main saturated fatty acid present in gum kondagogu. In addition, the researchers reported that the presence of mono- and polyunsaturated fatty acids such as palmitoleic acid (C16:1, 7.9 ± 0.54 lg/g), oleic acid (C18:1, 5.1 ± 0.18 lg/g), erucic acid (C22:1, 9.9 ± 0.54 lg/g), linoleic acid (C18:2, 1.8 ± 0.12 lg/g), linolenic acid (C18:3, 0.8 ± 0.05 lg/g) in gum kondagogu. The same researchers [31] investigated the fatty acid composition of gum karaya. They reported that the major fatty acids of gum karaya were capric acid (C10:0), lauric acid (C12:0), myristic acid (C14:0), palmitic acid (C16:0), palmitoleic acid (C16:1), stearic acid (C18:0), oleic acid (C18:1), and behenic acid (C22:0). The most abundant unsaturated fatty acids present in the chemical structure of gum karaya were palmitoleic acid (13.2 ± 0.95 lg/g), oleic acid (4.2 ± 0.21 lg/g), and erucic acid (2.8 ± 0.12 lg/g) [31]. The previous study [31] showed that gum karaya had higher content of SFA and lower content of PUFA than gum kondagogu. Initially, it was hypothesized that the fatty acid profile might be a reliable indicator to discriminate various plant gums from each other. However, the fatty acid composition of the gum is significantly affected by the extraction, purification and further processing condition; therefore this parameter cannot be an accurate tool to differentiate various plant gums from each other.

### Amino acid composition

**Figure 4:**
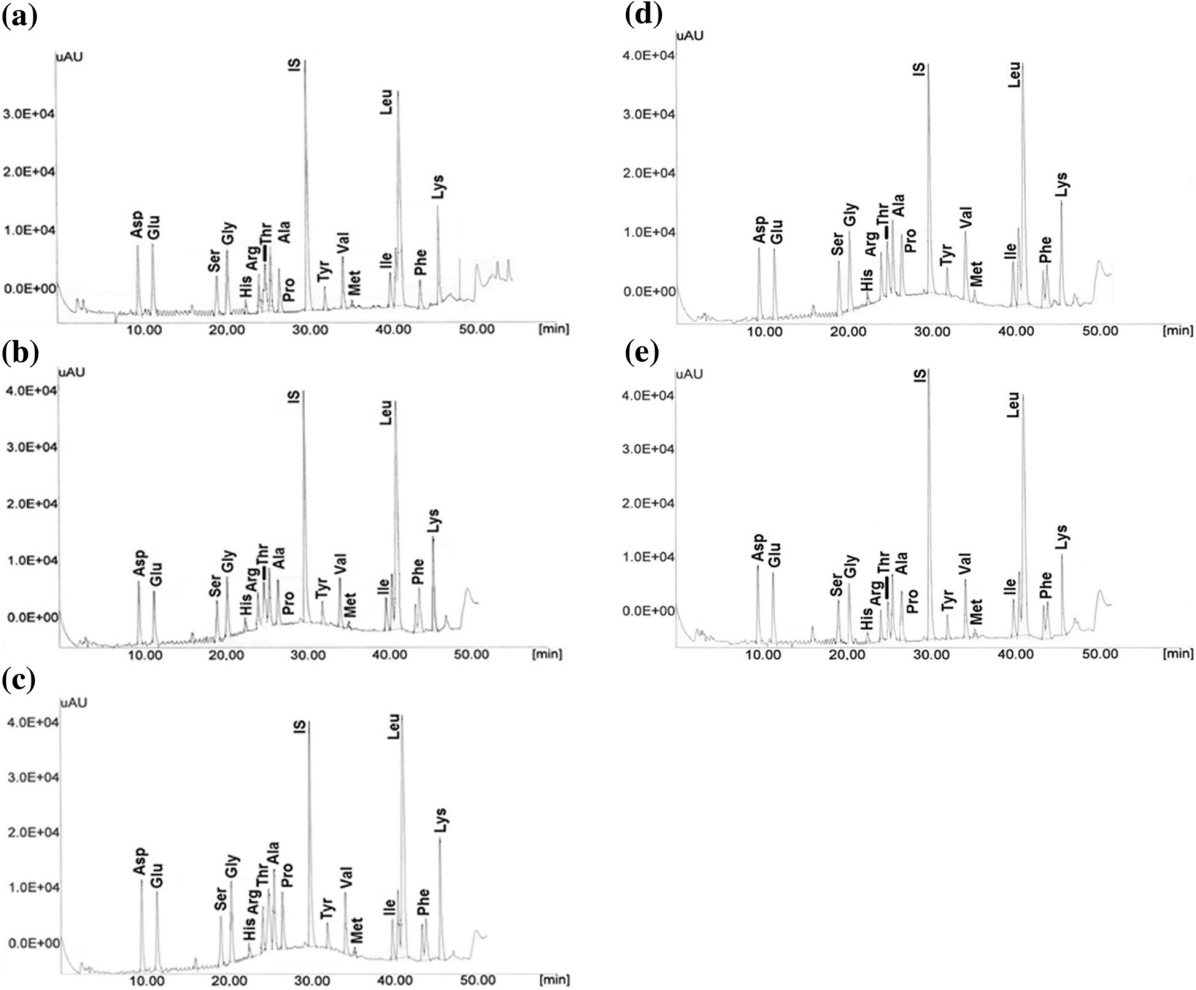
Amino acid profile of (a) crude gum, (b) purified gum a, (c) purified gum b, (d) purified gum c, (e) purified gum d; aspartic acid (Asp), glutamic acid (Glu), serine (Ser), glycine (Gly), histidine (His), arginine (Arg), threonine (Thr), alanine (Ala), proline (Pro), internal standard (IS, alpha amino butyric acid), tyrosine (Tyr), valine (Val), methionine (Met), isoleucine (Ile), leucine (Leu), phenylalanine (Phe), and lysine (Lys).

The amino acid composition can be a useful feature for the chemical characterization of the heteropolysaccharide gum. This parameter directly contributes to the solubility and functional properties of the heteropolysaccharide-protein gum. Our previous study [30] revealed the presence of the protein fraction in the chemical structure of durian seed gum. Figure 4 (a-e) demonstrates HPLC chromatograms which show the amino acid composition of the protein fraction present in the chemical structure of durian seed gum. The current study revealed that the most abundant amino acid present in the chemical structure of durian seed gum were leucine (Leu) (30.9-37.3%), phenylalanine (Phe) (3.11-9.04%), lysine (Lys) (6.04-8.36%), glycine (Gly) (6.07-7.42%), aspartic acid (Asp) (6.10-7.19%), glutamic acid (Glu) (5.57-7.09%), alanine (Ala) (5.24-6.14%), threonine (Thr) (3.44-6.50%), valine (Val) (4.5-5.50%), serine (Ser) (4.39-5.18%), proline (Pro) (3.87-4.81%), and isoleucine (Ile) (3.30-4.07%) (Table 5). In addition, the amino acid analysis also revealed the presence of a minor quantity of methionine (Met) (0.50-0.98%), histidine (His) (0.81-1.14%), arginine (Arg) (2.38-3.51%), and tyrosine (Tyr) (1.63-2.19%) in the amino acid composition of durian seed gum (Table 5). The presence of a negligible content of histidine, methionine and tyrosine was also reported by previous researchers in flaxseed gum [16], Spondias gums [32], and *Acacia glomerosa* gum [33]. They also reported different contents of leucine, phenylalanine, lysine, aspartic acid, glycine, alanine, glutamic acid, valine, proline, and serine in various plant gum such as flaxseed gum [16], Spondias gums [32], *Acacia glomerosa* gum [33], and Prosopis gum [34].

**Table 5:** Amino acid composition of the protein fraction from durian seed gum

**Amino acid**	**Control sample**	**Purified gum A**	**Purified gum B**	**Purified gum C**	**Purified gum D**
Asp	7.19 ± 0.13^a^	6.19 ± 0.44^b^	6.95 ± 0.26^ab^	6.10 ± 0.58^b^	6.50 ± 0.75^ab^
Glu	7.09 ± 0.22^a^	5.57 ± 0.25^b^	6.54 ± 0.34^a^	6.18 ± 0.35^ab^	6.42 ± 0.34^a^
Ser	4.67 ± 0.50^a^	5.06 ± 0.32^a^	4.53 ± 0.57^a^	5.18 ± 0.12^a^	4.39 ± 0.29^a^
Gly	7.42 ± 0.29^a^	6.22 ± 0.58^b^	6.43 ± 0.20^b^	7.36 ± 0.20^a^	6.07 ± 0.15^b^
His	1.13 ± 0.15^a^	1.14 ± 0.19^a^	0.88 ± 0.04^a^	1.00 ± 0.09^a^	0.81 ± 0.10^a^
Arg	3.51 ± 0.25^a^	3.40 ± 0.21^a^	2.87 ± 0.12^ab^	3.16 ± 0.16^a^	2.38 ± 0.22^b^
Thr	6.50 ± 0.19^a^	5.41 ± 0.42^b^	6.47 ± 0.45^a^	4.99 ± 0.43^bc^	3.44 ± 0.34^c^
Ala	6.14 ± 0.36^a^	5.68 ± 0.24^a^	5.97 ± 0.23^a^	6.08 ± 0.25^a^	5.24 ± 0.43^a^
Pro	4.35 ± 0.16^a^	4.17 ± 0.75^a^	3.87 ± 0.41^a^	4.81 ± 0.42^a^	4.06 ± 0.58^a^
Tyr	2.19 ± 0.21^a^	1.91 ± 0.09^a^	1.63 ± 0.22^a^	1.95 ± 0.19^a^	1.84 ± 0.29^a^
Val	5.15 ± 0.43^a^	4.59 ± 0.43^a^	4.50 ± 0.24^a^	5.50 ± 0.46^a^	4.90 ± 0.16^a^
Met	0.59 ± 0.07^a^	0.50 ± 0.03^a^	0.52 ± 0.06^a^	0.98 ± 0.10^b^	0.69 ± 0.09^a^
Ile	3.89 ± 0.18^a^	3.63 ± 0.22^a^	3.30 ± 0.29^a^	4.07 ± 0.23^a^	3.73 ± 0.22^a^
Leu	37.1 ± 1.87^a^	37.3 ± 1.67^a^	30.9 ± 1.87^b^	34.6 ± 1.87^ab^	35.6 ± 1.15^a^
Phe	3.11 ± 0.12^a^	9.04 ± 0.87^b^	6.93 ± 0.22^c^	8.13 ± 0.38^b^	7.62 ± 0.26^bc^
Lys	8.36 ± 0.23^a^	7.88 ± 0.46^a^	7.65 ± 0.65^ab^	7.32 ± 0.28^a^	6.04 ± 0.25^b^
Hpr	-	-	-	-	-

Aspartic acid (Asp), glutamic acid (Glu), serine (Ser), glycine (Gly), histidine (His), arginine (Arg), threonine (Thr), alanine (Ala), proline (Pro), tyrosine (Tyr), valine (Val), methionine (Met), isoleucine (Ile), leucine (Leu), phenylalanine (Phe), and lysine (Lys); A: isopropanol and ethanol; B: isopropanol and acetone; C: saturated barium hydroxide; D: Fehling solution; ^a-d^ significant at p < 0.05: Mean ± standard deviation.

Vinod and co-researchers [31] reported that the most abundant amino acids in gum kondagogu were Ala (32.2 ± 1.44 lg/g), Gly (5.05 ± 0.55 lg/g), Val (7.2 ± 0.60 lg/g), Leu (3.8 ± 0.22 lg/g), Pro (42.4 ± 2.56 lg/g), Met (44.2 ± 2.25 lg/g), Asp (72.8 ± 3.45 lg/g), Thr (30.4 ± 1.54 lg/g), Tyr (32.8 ± 1.85 lg/g), and Try (10.8 ± 0.84 lg/g). They also reported that the main amino acids in the chemical structure of gum karaya (lg/g) included Gly (4.8 ± 0.45 lg/g), Leu (3.9 ± 0.28 lg/g), Pro (30.5 ± 1.86 lg/g), Asp (64.2 ± 2.44 lg/g), Thr (25.2 ± 1.06 lg/g), and Glu (34.2 ± 1.44 lg/g). The most abundant amino acids were Asp (64.2 ± 2.44 lg/g) and Glu (34.2 ± 1.44 lg/g) [31]. The current study revealed that the amino acid composition of durian seed gum was different from that of reported for Spondias gum, Prosopis gum, gum kondagogu, flaxseed gum, gum karaya, gum Arabic, mesquite gum and guar gum [16,32-34]. For instance, previous researchers reported the presence of hydroxyproline (Hpr) in the chemical structure of Spondias gum, Acacia gum, Mesquite gum and Prosopis gum; while there was no evidence showing the presence of Hpr in the chemical structure of durian seed gum (Table 5).

### Molecular characteristics

The results showed that both extraction and purification processes significantly (p < 0.05) influenced the molecular structure of durian seed gum (Table 6). The results indicated that the molecular weight of crude and purified gum ranged from 1.08 × 10^5^ to 1.44 × 10^5^ (g/mol) (Table 6). As reported in our previous study [21], different purified durian seed gums showed significant (p < 0.05) different rheological behavior and viscoelastic properties. This might be due to the difference between the molecular weight of different crude and purified durian seed gums (A-D). As shown in Table 6, all purification processes led to reduce the molecular weight. Da Silva and Gonćalves [14] also showed that the purified locust bean gum (LBG) had lower molecular weight than the crude LBG. They reported that the purification of locust bean gum (LBG) using isopropanol led to reduce the molecular weight as compared to the crude LBG.

**Table 6:** **Molecular weight, and polydispersity index (M**_**w**_**/M**_**n**_**) of durian seed gum**

**Test**	**Crude gum (Aqueous)**	**Crude gum (Chemical)**	**Purified gum A**	**Purified gum B**	**Purified gum C**	**Purified gum D**
M_wt_ (g/mol)	1.37 × 10^5^ ± 0.024^a^	1.44 × 10^5^ ± 0.024^b^	1.26 × 10^5^ ± 0.015^c^	1.23 × 10^5^ ± 0.019^c^	1.14 × 10^5^ ± 0.026^d^	1.08 × 10^5^ ± 0.009^e^
M_w_/M_n_	1.34	1.39	1.29	1.26	1.21	1.17
R_g_ (nm)	28	31	25	23	18	15

M_wt_: Molecular weight; M_w_/M_n_: Molecular weight/number average molecular weight (or polydispersity index); A: isopropanol and ethanol; B: isopropanol and acetone; C: saturated barium hydroxide; D: Fehling solution.

The results indicated that the purification using isopropanol with ethanol (Method A) resulted in the least significant reduction in the molecular weight of durian seed gum (Table 6). On the other hand, the precipitation using Fehling solution (Method D) resulted in the highest significant (p < 0.05) reduction in the molecular weight of durian seed gum. In fact, the purified gum A and D had the highest and lowest molecular weight among purified samples (Table 6). This might be explained by the presence of NaOH in Fehling solution which probably enhanced the degradation of the polysaccharide structure. In addition, the copper complex present in Fehling solution may cause the cleavage in polysaccharide chains, thereby reducing the molecular weight of the purified gum D as compared to the crude gum. In addition, the removal of the lipid and/or protein fraction from the gum structure might be also responsible for the further reduction of the molecular weight during the purification process.

Different crude and purified durian seed gum had significant (p < 0.05) different Rg values ranging from 15 to 31 nm (chemically-extracted durian seed gum) (Table 6). The chemically-extracted durian seed gum and purified durian seed gum D showed the highest and lowest Rg values (31 nm and 15 nm), respectively (Table 6). The results indicated that the polydispersity index (Mw/Mn) of durian seed gum ranged between 1.17 and 1.39 (Table 6). The polydispersity index (PDI, Mw/Mn) of durian seed gum significantly decreased after the purification process. This might be interpreted by the significant reduction of molecular weight after purifying the crude gum. The purified gum with lower PDI has more homogeneous structure than the crude gum with higher PDI. The high polydispersity value for the crude durian seed gum might be due to the presence of molecules with a broad spectrum of molecular weights. The heterogeneous structure with a wide spectrum of molecular weights resulted in some difficulties in the precipitation process.

## Conclusions

The present study revealed that all purification processes led to reduce the lipid fraction. However, they did not completely eliminate the lipid fraction from the chemical structure of the gum. Among all purification techniques, the precipitation using Fehling solution induced the most significant (p < 0.05) changes in the chemical composition and molecular structure of the heteropolysaccharide gum from *Durio zibethinus* Murray seed. The purified gum D (using Fehling solution) had the least contents of total ash, soluble ash, and lipid fraction among all crude and purified gums. It seems that the purification using Fehling solution can provide the purified durian seed gum with the highest purity degree (or least impurity content) among all studied purification techniques. The present study revealed the presence of some essential mono- and polyunsaturated fatty acid (e.g. oleic acid, linoleic acid, and linolenic acid) in the chemical structure of durian seed gum. The presence of essential amino acids (i.e. phenylalanine, valine, tryptophan and isoleucine) in the chemical structure of durian seed gum reinforces its nutritional value as compared to amino acid free gums. The present study recommends testing the biological and nutritional aspects of the natural biodegradable biopolymer from durian seed.

### Experimental

#### Chemicals and materials

The sugar standards (i.e. L-arabinose, D-(+)-galactose, D-(+)-mannose, L-rhamnose monohydrate, D-(+) glucose, D-(−)-fructose and sucrose) were purchased from Sigma-Aldrich (St. Louis, MO, USA). Acetonitrile (HPLC grade), sulfuric acid (nitrogen free), ammonium acetate, sodium hydroxide (NaOH) and hydrochloric acid (HCl) (37%) were supplied by Merck (Darmstadt, Germany). In this study, amino acid standards AAS18 including L-alanine (Ala), L-glycine (Gly), L-valine (Val), L-leucine (Leu), L-isoleucine (Ile), L-threonine (Thr), L-methionine, (Met), L-aspartic acid (Asp), L-glutamine (Gln), L-phenylalanine (Phe), L-tyrosine (Tyr), L-tryptophan (Trp) and hydroxyproline (Hpr) were purchased from Sigma-Aldrich (St. Louis, MO, USA). Ethanol 95%, absolute ethanol (99.9%), saturated barium hydroxide, acetic acid, hexane, petroleum ether (40–60°C) and methanol (HPLC grade) were purchased from Fisher Scientific (Pittsburgh, PA, USA). Durian (*D. zibethinus*) fruit was purchased from the local market (Selongor, Malaysia). Ripened durian fruits were selected based on the size uniformity and free of visual defects. The fruits were then de-husked (cut open the rind), by cutting along the suture on the back of the lobules. Durian seeds were removed, cleaned and rinsed thoroughly with sterile distilled water. The seed was partially dried by the air circulation. The dried seeds were then packed in plastic bags and stored in a dry and cool place (10 ± 2°C) until the extraction process. All the experiments were performed with deionized water [35].

### Extraction process

#### Aqueous extraction

The aqueous extraction of the crude gum from durian seed was carried out in triplicate according to the method described earlier [35]. In this method, durian seeds were washed, chopped and ground into the powder form. Then, the aqueous extraction process was performed by using deionized water from durian seed powder. The aqueous extraction was carried out by using water: seed (W/S) ratio of 60:1, extraction temperature 40°C under pH 7. In this experiment, pH was continuously adjusted by 0.1 (mol/L) NaOH and HCl. The heat treatment was performed indirectly by using an adjustable water bath. It avoids the thermal degradation of the sample which may be occurred by the direct heat treatment. In fact, the water temperature of the adjustable water bath was controlled before the seed powder was added to the beaker. The seed-water slurry was stirred throughout the entire extraction period (1 h, based on our preliminary tests). The slurry was centrifuged at 1200 rpm for 10 min by using a Beckman Coulter Centrifuge (Avanti J-25, Beckman Coulter GmbH, Krefeld, Germany). Subsequently, the supernatant was collected. The supernatant was mixed with three volumes of 95% ethanol and the percipient was dispersed in deionized water and oven dried at 40°C [35].

#### Chemical extraction

The chemical extraction was performed according to the method described in the previous study [36]. Durian seed were washed and chopped into small pieces. Then, it was air dried by using the air circulation before milling into flour. The cold extraction was used to extract the oil from durian seed flour in order to avoid the thermal degradation. The defatting process was carried out successively using hexane and isopropanol (60:40) at the room temperature (25 ± 1°C). Our preliminary study showed that the solvent mixture containing hexane and isopropanol (60:40) was the most efficient solvent for defatting process among all studied solvents (i.e. petroleum ether, hexane, isopropanol and ethanol). The solvent residue was removed by centrifugation at ~3000 rpm for 15 min using the Beckman Coulter Centrifuge (JA-14, Beckman Coulter GmbH, Krefeld, Germany). Then, defatted-durian seed flour (1 kg) was exhaustively decolored using ethanol at the decoloring time 120 min. The decolorized seed flour was vacuum filtered and then soaked in 1% aqueous acetic acid for 1.5 h at the room temperature (25 ± 1°C). Then, the slurry was filtered with Nylon cloth filter and the filtrate was precipitated with 95% ethanol. The precipitated slurry was washed three times using absolute ethanol (99.9%) to achieve very light brown amorphous crude gum. The crude gum was collected and oven dried at 40°C [36]. The effectiveness of four different purification techniques was determined by considering the crude seed gum as a control sample.

#### Purification process

In the purification Method A, the crude seed gum was purified by using hot water, ethanol and isopropanol as described by previous researchers [19,37]. Initially, the gum solution (2.5% w/v) was prepared by dissolving 25 g of the crude durian seed gum in 1 l of deionized water at 80°C water bath for 6 h, followed by stirring at room temperature overnight. The gum solution (2.5% w/v) was subjected to the centrifugation for 15 min at ~10000 rpm using the Beckman Coulter Centrifuge (JA-14, Beckman Coulter, Krefeld, Germany). The supernatant was precipitated by the addition of absolute ethanol (1.2 l). Then, the supernatant was decanted, and the residue was recovered and kept overnight in 100% isopropanol. Finally, the residue was dried in the oven 40°C for overnight to prepare the purified durian seed gum.

In the purification Method B, the purification process was carried out by using isopropanol and acetone as reported by previous researchers [38] with the minor modification. One g of the crude seed gum was precipitated by soaking into 200 ml isopropanol, and allowing the gum-solvent slurry to stand for 30 min. The fibrous precipitate was collected by the filtration using screen with mesh 53 μm. Then, the collected precipitate was washed twice with isopropanol and acetone [21]. Finally, it was dried in the oven overnight at 40°C.

In the purification Method C, the crude seed gum was purified through barium complexing according to the method described by previous researchers [39]. In this method, the gum solution (2.5% w/v) was prepared by dissolving 2.5 g of the crude durian seed gum in 100 ml of water and continuous stirring for 12 h at 60°C. Then, the gum solution was precipitated with saturated barium hydroxide solution. The precipitate was separated by the Beckman centrifuge at 3500 rpm for 15 min. Then, the precipitate was stirred with 1 M acetic acid for 8 h and again centrifuged. The supernatant was precipitated with 90% ethanol. Finally, the precipitate was washed with 95% ethanol and oven dried at 40°C [37].

In the purification Method D, the purification was performed by using Fehling solution as reported by previous researchers [20] with some modification. Initially, 1 g of the crude durian seed gum was dissolved in approximately 100 ml of water and stirred for 24 h with magnetic stirring. The prepared gum solution (1% w/v) was precipitated by adding 5 ml of freshly prepared Fehling solution, and the precipitate was collected on a glass filter (No. 3). Then, the precipitate was dissolved in 0.1 M hydrochloric acid while under magnetic stirring for 1 h until the full solubilisation. The solution was precipitated with 95% ethanol (120 ml). The precipitate was separated by the glass filter (No. 3) and washed with 95% ethanol until pH 6 [37]. Finally, the filtrate was washed with acetone and oven dried at 40°C overnight.

### Analytical test

#### Determination of sugar composition

The sugar composition was determined according to the method reported by Amin and co-workers [6] with minor modifications. D-fructose was considered as an internal standard in the present study. The carbohydrate profile was determined by using a high performance liquid chromatography (HPLC) (Waters 486, CA, USA) equipped with a refractive index (RI) detector and a pump Waters 600 controller. The analytical column was Lichrocart 250–4,6 purospher star NH_2_ column (5 MYM). The mobile phase composed of acetonitrile–water (75:25). The flow rate was ranged between 0.4–1.5 ml/min. A 10 mg of crude durian seed gum was degraded by heating at 80°C for 24 h along with 2 ml of 1 M sulfuric acid. Next, the hydrolyzed gum was placed under the rotary evaporator for 4 h at 40°C under the vacuum condition. Then, 1 ml of deionized water was added to the remaining gum solution. Finally, the slurry was filtered by using Sep-Pak cartridges (Waters Associates, Milford, MA, USA) to remove the phenolic compounds. The filtrated slurry was passed through a membrane filter of 0.45 μm (Whatman) before injecting to HPLC system. The sugar analysis was carried out in triplicate for each sample. For quantitative analysis of the sugar composition, the following standards were considered: L-arabinose, D-galactose, D-mannose, D-xylose*,* L-rhamnose, glucose, fructose (IS) and sucrose.

#### Moisture content

The moisture content was determined by using the method described by previous researchers [6]. Approximately, 2 g of durian seed gum was weighed in the crucible and then placed in the oven at 100°C for 6 h. After drying at 100°C, the dried sample was covered, cooled in the desiccator and weighted, accordingly. The gum samples were taken out and weighed after every 10 min interval time. This was repeatedly done until the weight of the samples remained constant. The moisture content was measured in triplicate and estimated based on the following equation:

(1) $$\begin{array}{l} \%\;\text{Dry}\;\text{Content}=\frac{\text{Weight}\;\text{of}\;\text{dry}\;\text{sample}}{\text{Original}\;\text{weight}\;\text{of}\;\text{sample}}\times 100\\ \%\;\text{Moisture}\;\text{content}=100-\%\;\text{dry}\;\text{content} \end{array}$$

#### Ash content

The total ash was determined according to AOAC Official Method 923.03. [40]. Approximately 5 g of durian seed gum was weighed into a shallow ash dish. The dish containing the gum was ignited, cooled in desiccator and weighed after cooling to the room temperature. Then, the sample was ignited in a furnace at 550°C (dull red) until the weight become constant. In this condition, light gray ash was observed in the dish. Then the gray ash was weighed after the sample was cooled to the room temperature in the desiccator. The total ash content was calculated according to the following equation:

(2) $$\%\;\text{total}\;\text{ash}=\frac{\text{ash}\;\text{weight}}{\text{original}\;\text{sample}\;\text{weight}}\times 100$$

Soluble ash content was determined by mixing the total ash with 25 ml distilled water, and the solution was heated to boil. Then the solution was filtered and soluble ash was rinsed using the distilled water until the volume was about 60 ml. The filter paper and its residue were placed back into the original crucible, where the dish containing the gum was ignited in this step. The crucible was placed in the oven again at 550°C until the constant weight, then cooled and weighed. The ash content was measured in triplicate for each sample. The soluble ash was calculated according to the following equation:

(3) %solubleash=%totalash-%insolubleash

#### Lipid content

The lipid extraction was carried out by hydrolyzing durian seed gum according to the method reported by previous researchers [41,42] with minor modification. For hydrolysis purpose, 1 g durian seed gum was placed in a screw cap glass tube (55mL, 25mm × 150mm), and then 10 ml of 1.5 M methanolic KOH and 500 μl water were added to dissolve the sample. The tubes were sealed with Teflon lined screw caps. Subsequently, the tubes were immersed in a water bath at 70°C for 1 h under stirring mode. The tubes were gradually cooled to the room temperature. Then, 6 ml methanol and 8 ml chloroform were added to the tubes and mixed well. The tubes were subjected to the centrifugation at 70 × g for 15 min and filtered through a Whatman GF/A glass filter paper (Whatman Laboratory Products, Clifton, NJ, USA) fitted in a Buchner funnel under the vacuum condition. The pellet in the glass tube was suspended in 2 ml of methanol and chloroform (2:1). The tube was mixed well and filtered through the same filtration set up to collect the filtrate in the same tube. The filter paper was rinsed with 1 ml of 2:1 methanol and chloroform (2:1) and the filtrate was collected in the same tube. Subsequently, 8.5 ml of water was added to the filtrate containing 18 ml methanol and 9 ml chloroform. Then, 6 M HCl was used to acidify the solution (pH 2–3) and 9 ml chloroform was added to the solution to maintain the ratio of methanol, chloroform and water (2:2:1) and help the phase separation [43]. The reaction mixture was vortexed and centrifuged at 70 × g for 10 min for full phase separation. The lower layer (i.e. chloroform layer) was collected in a clean vial. The residue was weighed after evaporating the solvent at 50°C under the pure nitrogen stream. The lipid content was measured in duplicate for each sample.

#### Fatty acid composition

Fatty acid composition of lipid fraction from durian seed gum was analyzed by using gas chromatography (Hewlett–Packard GC 6890, Chicago, USA) equipped with a flame ionization detector (FID) and a fused silica capillary DB-Wax column (30 mm × 0.32 mm i.d. × 0.25 lm film thickness) (Agilent Technologies, Chicago, USA). Fatty acid methyl esters were prepared by using sodium methoxide and methanol [44]. The injection condition was split mode with the split ratio of 1:10 and injection volume of 2 μl sample. The injector port and detector temperature were set at 240°C and 270°C, respectively. Helium was used as a carrier gas with the flow rate of 0.4 ml/min under the constant flow. The oven temperature was initially set at 80°C, subsequently raised to 170°C at the flow rate of 10°C/min and kept for 5 min at 170°C. The temperature was subsequently raised to 210°C at the flow rate of 2°C/min and held for 2 min. Finally, it was raised up to 240°C at the flow rate of 5°C/min and kept for 15 min at 240°C. Peak retention times and area percentages of total fatty acids were determined by injecting known amount of FAME standards, and subsequently analyzed with the Agilent Technologies ChemStation A.08.03 software. The fatty acid analysis was performed in triplicate for each sample.

#### Amino acid analysis

For amino acid analysis, 0.25 g durian seed gum was weighed and hydrolyzed by using 15 ml 6 N HCl. Then, it was mixed in a test tube and kept for 24 h at 110°C. The hydrolysate was dried under the vacuum condition. The derivatization was carried out by using phenylisothiocyanate. In this experiment, buffer A (0.1 M ammonium acetate, pH 6.5) and buffer B (0.1 M ammonium acetate, acetonitril, and methanol, 44:46:10 v/v, pH 6.5) were used as mobile phases. For HPLC analysis, 40 μL of the sample containing the mobile phase A was injected into the HPLC system equipped with Photodiode Array Detector (model MD-2010; JASCO, Tokyo, Japan) and reversed phase column RP-C18 (LICHROCART 250–4,6, 250 × 5 mm) (Merck, Darmstadt, Germany). Alpha amino butyric acid (AABA) was used as an internal standard (IS). The linear gradient system was used at the flow-rate of 1 ml/min in an oven at 40°C. The UV absorption detection at a wavelength of 254 nm was applied to detect the content of amino acids. The amino acid analysis was performed in triplicate for each sample. The result was analyzed by using JASCO Borwin chromatography software (V. 1.5, Jasco Co. Ltd., Japan).

#### Molecular weight

In the present study, size-exclusion chromatography coupled to multi angle laser light-scattering (SEC-MALS) system was applied to measure the molecular weight according to the method reported by previous researchers [45]. It should be noted that the multi angle laser light scattering (MALLS) is for the determination of molecular weight over broad ranges. The system was equipped with one guard column (TSK-G), a JASCO PU-980 HPLC pump (Tokyo, Japan) and two separation columns (TSKgel G-6000 PWxL and TSKgel GMPWxL) (Tosoh Co., Tokyo, Japan). Exclusion limits of the separation columns were both 50.0 × 10^6^ g/mol on a dextran base. Air bubbles of the aqueous solutions containing 0.05 M NaNO3 were removed by using an on-line degasser JASCO DG-980-50. Detectors were calibrated by using filtered toluene and normalized with pullulan (23.8 K) (Polymer Standards Services GmbH, Mainz, Germany). A circulating flow rate of 0.5 ml/min was applied in the system. Static light-scattering measurements using a DAWN-DSP (Wyatt Technology Co., CA, USA) were carried out at 25°C, and scattering intensity was determined at angles from 261 to 1321 concurrently with multiple detectors. In this experiment, 0.5 g durian seed gum was used to prepare the gum solution (0.05%), and the solution was filtered through ADVANTEC cellulose-acetate membrane filters (0.45 mm pore size). Finally, 100 μl of the solution (0.05%) was injected to the system after filtering through the cellulose-acetate membrane. The molecular weight was calculated based on the following equations [46]:

(4) $$\frac{KC}{R_{\theta }}=\frac{1}{M_{w}}\left[1+16\pi^{2}\langle r_{g}^{2}\rangle \sin^{2}\left(\theta \right)/3\lambda^{2}\right]+2{\text{A}}_{2}\text{C}$$

(5) $$K=4\pi^{2}n_{o}{\left(\text{d}n/\text{d}c\right)}^{2}/\lambda^{4}N_{\text{A}}$$

(6) $$M_{w}=\frac{\sum \left(c_{i}M_{i}\right)}{\sum c_{i}}$$

(7) $$M_{w}=\frac{\sum c_{i}}{\sum \frac{c_{i}}{M_{i}}}$$

(8) $${\langle r^{2}\rangle }_{z}=\frac{\sum \left(c_{i}M_{i}{\langle r^{2}\rangle }_{i}\right)}{\sum \left(c_{i}M_{i}\right)}$$

*K* is an optical constant, *R*_*θ*_ is the excess Rayleigh ratio which is the measured quantity, *θ* is the scattering angle, *M*_w_ is the average molecular weight, *A*_2_ is the second viral coefficient, and *λ* is the wavelength of light. The quantities *c*_*i*_, Mn M_w_ are the concentration, number average molecular weight, and molecular weight, respectively [46]. The ratio of Mw/Mn represents the polydispersity index. The measurement was triplicate for each sample and the average of three measurements was reported for further data analysis.

#### Experimental design and data analysis

The effect of different extraction and purification methods on the chemical and molecular structure of durian seed gum was investigated by using the completely randomized design (CRD). Four different purification methods (namely Method A (isopropanol and ethanol), Method B (isopropanol and acetone), Method C (saturated barium hydroxide) and Method D (Fehling solution)) were chosen based on our previous study and literature [19,21,37,39]. The sugar composition, moisture, ash, lipid content, fatty acid composition, molecular weight (M_w_), and other parameters (M_w_/M_n_ and R_g_) related to the molecular structure of durian seed gum were determined as response variables. The performance of different extraction and purification techniques was determined by comparing the chemical and molecular structure of different crude and purified gums. The data was subjected to one way analysis of variance (ANOVA) to determine the significant (p < 0.05) differences among the samples. All data analysis was carried out by using Minitab version 15 (Minitab Inc., PA, USA). Fisher multiple comparison test was used to evaluate significant differences (p < 0.05) between the different purified seed gums as compared to the control.

## Abbreviations

AABA: Alpha amino butyric acid; ANOVA: One way analysis of variance; AOAC: Association of Official Analytical Chemists; CRD: Completely randomized design; D: *Durio*; FAME: Fatty acid methyl ester; FID: flame ionisation detector; GF: Glass filter; HPLC: High performance liquid chromatography; i.d: Inner diameter; IS: Internal standard; LBG: Locust bean gum; Molar: M; M_w_: Molecular weight; Mw/Mn: Molecular weight/number average molecular weight; RP-C: Reversed phase column; SEC-MALS: Size-exclusion chromatography coupled to multi angle laser light-scattering; UV: Ultra violet; v/v: Volume/volume; W/S: Water: seed.

## Competing interests

The authors declared that they have no competing interest.

## Authors’ contributions

BA carried out all the experiments and data analysis. BA also prepared the drafted manuscript, and all authors read, edited and approved the final manuscript.
